# Comparative effect of Tai Chi and aerobic exercise on cognitive function in advanced lung cancer survivors with perceived cognitive impairment: a three-arm randomized controlled trial with mediation analysis

**DOI:** 10.1007/s11764-024-01607-1

**Published:** 2024-05-01

**Authors:** Naomi Takemura, Denise Shuk Ting Cheung, Daniel Yee Tak Fong, Anne Wing Mui Lee, Tai-Chung Lam, James Chung-Man Ho, Tsz Yeung Kam, Jeannie Yin Kwan Chik, Chia-Chin Lin

**Affiliations:** 1 School of Nursing, Li Ka Shing Faculty of Medicine, The University of Hong Kong, Pokfulam, Hong Kong; 2 Department of Clinical Oncology, The University of Hong Kong-Shenzhen Hospital, Guangdong, China; 3 Department of Clinical Oncology, Li Ka Shing Faculty of Medicine, The University of Hong Kong, Pokfulam, Hong Kong; 4 Department of Medicine, Li Ka Shing Faculty of Medicine, The University of Hong Kong, Pokfulam, Hong Kong; 5 Department of Clinical Oncology, Pamela Youde Nethersole Eastern Hospital, Chai Wan, Hong Kong; 6 Department of Clinical Oncology, Queen Elizabeth Hospital, Kowloon, Hong Kong; 7 Alice Ho Miu Ling Nethersole Charity Foundation Professor in Nursing, Pokfulam, Hong Kong

**Keywords:** Aerobic exercise, Tai Chi, Cognitive function, Randomized controlled trial, Lung cancer

## Abstract

**Purpose:**

Cancer-related cognitive impairment is prevalent in metastatic lung cancer survivors. This study aimed to compare the effectiveness of aerobic exercise and Tai Chi on perceived cognitive function and the mediating role of psychoneurological symptoms with perceived cognitive impairment.

**Methods:**

In a subgroup of a parent randomized clinical trial, participants who reported cognitive impairment underwent a 16-week aerobic exercise (*n* = 49), Tai Chi (*n* = 48), and control (*n* = 54) groups. Measures included perceived cognitive function and psychoneurological symptoms (sleep disturbance, fatigue, anxiety, and depression) assessed at baseline (T0), 16-week (T1), and 1 year (T2).

**Results:**

Participants in Tai Chi showed significant improvements compared to aerobic exercise and control groups in perceived cognitive function at T1 (AE: between-group difference, 6.52; *P* < 0.001; CG: 8.34; *P* < 0.001) and T2 (AE: between-group difference, 3.55; *P* = 0.05; CG: 5.94; *P* < 0.001). Sleep disturbance, fatigue, anxiety, and depression at month 12 explained 24%, 31%, 32%, and 24% of the effect of the intervention on cognitive function at month 12, respectively. Only anxiety at month 4 explained 23% of the intervention effect at month 12.

**Conclusions:**

Tai Chi demonstrated beneficial effects on cognitive function in advanced lung cancer survivors with perceived cognitive impairment. Improvement in cognitive function was mediated by reducing sleep disturbance, fatigue, anxiety, and depression, highlighting the importance of addressing these symptoms in future interventions to improve cognitive function, with anxiety playing a significant role at an earlier stage.

**Implications for Cancer Survivors:**

Tai Chi is a potentially safe complementary therapeutic option for managing cognitive impairment in this vulnerable population.

**Trial registration:**

ClinicalTrials.gov identifier: NCT04119778; retrospectively registered on 8 October 2019.

## Introduction

Cancer-related cognitive impairment is prevalent in patients with metastatic non-small cell lung cancer, with a prevalence of 30–95% [1–8]. Deterioration in cognitive function is a common side effect of cancer diagnosis that persists after treatment completion [9], with up to 30% of patients experiencing cognitive function deterioration before treatment, 75% during treatment, and 35% experiencing it for up to a decade after treatment [10]. Cancer-related cognitive impairment is observed in memory, attention, concentration, information processing speed, and executive function [11]. These changes in cognition can negatively impact quality of life, social roles, and even survival [12]. The proposed mechanisms underlying the pathophysiology of cancer-related cognitive impairment include direct effects on the central nervous system and indirect effects related to the immune response of the body to cancer and its treatment or regulatory issues with the hypothalamic–pituitary–adrenal axis, which could affect the brain hormone concentrations [13, 14]. The direct and indirect effects on cancer-related cognitive impairment may be aggregated by a psychonuerological response to a life-threatening illness such as sleep disturbance, fatigue, anxiety, and depression [15, 16].

Given the high prevalence of cancer-related cognitive impairment and its associated side effects, effective treatment options remain elusive. Pharmacological methods targeting the presumed mechanisms, such as inflammation and oxidative stress for cancer-related cognitive impairment, have unsuccessfully been researched [11, 17]. Therefore, increasing attention has been focused on non-pharmacological interventions, namely, physical activity, to alleviate cancer-related cognitive impairment [13, 18]. The promising role of physical activity in improving cognitive function has been demonstrated in older adults [19], in those with mild cognitive impairment [20], and in severe neurocognitive impairment [21]. Hence, interest has arisen in the potential of physical activity as an effective intervention strategy for cancer-related cognitive impairment.

Multiple mechanisms may underlie the effects of physical activity on cognitive function [10]. Engaging in physical activity can mitigate cognitive decline by increasing the production of neurotransmitters such as dopamine and norepinephrine, stimulating the production of neurotrophic factors such as brain-derived neurotrophic factor [22], and reducing inflammatory marker levels [23]. Additionally, all these mechanisms collectively alleviate the effects of psychoneurological symptoms commonly experienced by patients with cancer, such as fatigue, stress, sleep problems, anxiety, and depression, which may mediate the effects of physical activity on cognitive function [24, 25]. Aerobic and mind–body exercises are two commonly researched modalities of physical activity that promote physical and psychological well-being in patients with cancer [26]. However, evidence supporting the effect of physical activity on cancer-related cognitive impairment remains preliminary [18], and no studies have compared the effects of aerobic and mind–body exercises on cognitive function. Apart from examining the cognition-promoting effects of physical activity, studies identifying the mediating mechanisms underlying the relationship between physical activity and cognitive function are lacking. Addressing this gap is crucial for understanding the cognitive benefits of physical activity, hence providing insights into developing physical activity interventions that can advance changes in the mediators.

Therefore, the primary aim of this study was to compare the effects of aerobic exercise and Tai Chi, a well-known mind–body exercise, on the cognitive function of advanced lung cancer survivors with perceived cognitive impairment. Additionally, this study examined whether multiple psychoneurological symptoms (sleep disturbance, fatigue, anxiety, and depression) mediate the effects of physical activity on cognitive function. Compared to the control group, we hypothesized that aerobic exercise and Tai Chi would significantly benefit cognitive function and that the effect of physical activity on cognitive function would be mediated by improved sleep, fatigue, anxiety, and depression.

## Methods

### Design

The dataset was a subset from a multi-centered, three-arm randomized controlled trial comparing aerobic exercise, Tai Chi, and self-managed control groups on subjective sleep quality and other associated bio-physio-psychological outcomes in patients with advanced lung cancer (*n* = 226). This study is exploratory in nature, conducted as a secondary data analysis. The design, methods, and principal results of this trial, without the perceived cognitive function described herein, have been reported previously [27]. The study was approved by the Institutional Review Board of The University of Hong Kong/Hospital Authority Hong Kong West Cluster (UW 18–154), Hong Kong East Cluster (HKECREC-2019–014), and Kowloon Central Cluster/Kowloon East Cluster (KC/KE-19–0039/ER-3) and conducted following the Declaration of Helsinki. All participants provided written informed consent, and data anonymity was ensured. The trial followed the Consolidated Standards of Reporting Trials (CONSORT) guidelines.

### Study participants and randomization

Adult patients diagnosed with stage IIIB or IV non-small cell lung cancer without other cancer diagnoses and scored at least 2 (i.e., at least a little) in at least one item in the cognitive functioning scale of the European Organization for Research and Treatment of Cancer Quality of Life Core Questionnaire (EORTC QLQ-C30) [7] were eligible for this study. Exclusion criteria included an Eastern Cooperative Oncology Group Performance Status score of > 2 and regular exercise habits. Enrolled participants were randomly allocated to aerobic exercise, Tai Chi, and self-managed control groups at a 1:1:1 ratio using computer-based permuted block randomization with block sizes randomized between three, six, and nine. Randomization was stratified according to primary cancer treatment (targeted or non-targeted therapy). The outcome assessors were blinded to the group allocation.

### Interventions

#### Aerobic exercise class

For 16 weeks, the aerobic exercise intervention group received eight supervised group exercise sessions (two sessions per month) and home-based exercises. To accommodate any missed classes, one make-up session was arranged per month. Each supervised session lasted an hour and was conducted by two certified exercise specialists. Each session included aerobic and muscle-strengthening exercises with a moderate-intensity level, as indicated by a rating of 3–4 on the rating of perceived exertion (range: 0–10). For home-based exercises, participants were encouraged to perform at least 150 min of moderate-intensity aerobic exercise weekly and 2–3 sets of resistance exercises every other day. WhatsApp messages were provided weekly to encourage the participants to continue the exercise. The participants were provided exercise diaries to record their exercise type, frequency, and intensity. Printed materials and videos were delivered to the participants, covering the details of the prescribed weekly exercises to ensure postural consistency.

#### Tai Chi class

For 16 weeks, the Tai Chi intervention group received a twice-weekly 60-min 16-form Yang-style Tai Chi exercise session, which is the most popular traditional Tai Chi style [28]. The classes were all taught by an experienced and qualified Tai Chi master, who was the seventh-generation inheritor of Yang style Tai Chi and had accumulated over 10 years of experience teaching Yang style Tai Chi. The Tai Chi class protocol has been published in our parent trial [27]. Each class comprised standing meditation, mindful breathing exercises (warm-up), Tai Chi movements, and cool down stretching. Participants were given exercise diaries to record their exercise patterns, and videos of the Tai Chi movements taught in each class were delivered after class. A DVD containing all 16 forms of Tai Chi movements was delivered to the participants to facilitate self-practice at home.

Study investigators and research assistants monitored both intervention classes to ensure the participants’ safety and instructors’ adherence to the exercise programs.

#### Self-managed control group

Participants in the self-managed control group received written information on the recommended level of physical activity from the World Health Organization (i.e., at least 150 min of moderate-intensity or 75 min of vigorous-intensity aerobic physical activity throughout the week) [29], in addition to the usual care they received from the hospital. Participants were provided with exercise diaries to record their exercise patterns.

### Outcome measures

Measurements were obtained from all participants at three time points: baseline (T0), post-intervention (week 16; T1), and 8 months post-intervention (T2).

The primary outcome of this analysis—perceived cognitive function—was measured using the EORTC QLQ-C30 cognitive functioning subscale [30], which includes two items that assess perceived concentration and memory impairments. Each item is rated from 1 (not at all) to 4 (very much). The EORTC QLQ-C30 cognitive function subscale was also used to assess perceived cognitive impairment in patients with cancer [18]. The subscale and individual item scores were transformed into a 0 to 100 scale according to the EORTC scoring instructions, with higher scores indicating better cognitive functioning.

Psychoneurological outcomes included (1) subjective sleep quality as measured by the validated Pittsburgh Sleep Quality Index (Chinese version) [31], (2) anxiety and depression subscales as measured by the validated Chinese version of the Hospital Anxiety and Depression Scale [32], and (3) fatigue as measured by the validated Chinese version of the Brief Fatigue Inventory [33]. Sociodemographic variables (age, sex, education, and marital status), cancer-related information (current treatment modalities (targeted or non-targeted therapy), time since diagnosis (months)), and lifestyle factors (smoking (smoker or non-active smoker) and drinking (drinker or non-active drinker)) were assessed using a self-designed questionnaire. Nurses assessed the Karnofsky Performance Status score [34], measuring patient activity and independence.

### Statistical analyses

The study was powered for the primary endpoint of subjective sleep quality, and details of the sample size calculation have been previously reported [27]. Descriptive statistics were used to summarize the sociodemographic and medical variables of the participants at each time point. Baseline characteristics between the two interventions and control groups were compared using the *χ*2 test or Fisher’s exact test and analysis of variance (ANOVA) for categorical and continuous data, respectively. The intention-to-treat principle was used, with all participants analyzed as randomized and included participants who had at least one outcome measurement taken. A mixed-effects linear regression was used, with the intercept taken as random, and time, group, primary cancer treatment, and group × time interaction included as independent variables to assess the intervention effects on the primary and secondary outcomes. Linear contrasts were used to obtain (1) between-group differences and (2) within-group change from the baseline. Multiplicity due to multiple comparisons among the three groups at the two follow-up time points was accounted for using the Bonferroni approach. The normality of the residuals and random effects was assessed using skewness and normal probability plots. Missing values were not replaced because the mixed-effects model could accommodate participants with at least one outcome measurement [35].

The PROCESS macro for SPSS 28.0 [36] was used to examine the mediating effects of psychoneurological symptoms (sleep disturbance, fatigue, anxiety, and depression) on the relationship between attending exercise interventions and changes in cognitive function. We determined the mediation extent of the 4- and 12-month psychoneurological symptoms on 12-month cognitive function. Total, direct, and indirect effects were extracted, and the mediation percentage was determined. In all the models, the baseline value of the outcome was included as a covariate. Bias-corrected bootstrapping with 5000 replications was used to generate 95% confidence intervals for the direct and indirect effects. The effect was considered statistically significant if the 95% CI did not include zero.

## Results

**Fig. 1:**
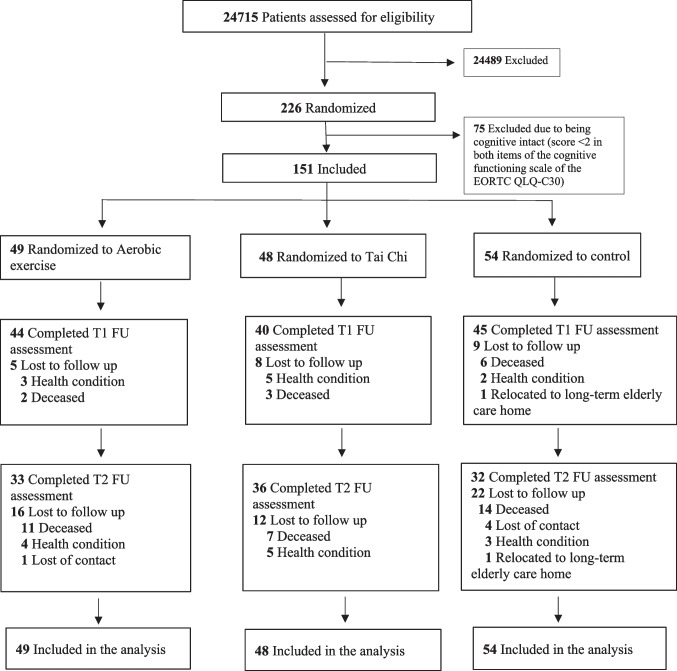
CONSORT 2010 flow diagram. Health condition refers to the worsening of a participant’s condition such that they are unable to continue with the intervention or follow-up assessments

The sample available for the current analysis was comprised of 151 participants (49 in the aerobic exercise group, 48 in the Tai Chi group, and 54 in the control group) (Fig. 1). The dropout rates, including the deceased, were 22 (14.6%) at T1 and 50 (33.1%) at T2. The mean (SD) attendance rates for aerobic exercise and Tai Chi were 8.24 (1.80) and 26.94 (7.81), respectively. The mean (SD) self-practice duration per week during the intervention period of the aerobic exercise, Tai Chi, and control group was 140.51 (58.33), 107.92 (65.16), and 37.89 (48.05) minutes, respectively.

### Baseline characteristics of participants

The baseline characteristics of the study participants are presented in Table 1. The mean age of the participants was 60.72 years (range: 35–78 years), and 57.6% were female. The mean Karnofsky Performance Status score was 89.14, and the mean time since diagnosis was 26.70 months. The aerobic exercise, Tai Chi, and control groups had similar baseline sociodemographic and clinical variables. Overall, 53% and 87% of the participants self-reported experiencing difficulty concentrating and remembering things, respectively.

**Table 1:** Baseline characteristics of the participants (*n* = 151)

Characteristic	Participants, no. (%)				*P*-value
	All (*N* = 151)	Aerobic exercise (*n* = 49)	Tai Chi (*n* = 48)	Control (*n* = 54)	
Age, mean (SD), y years	60.72 (8.72)	60.67 (7.76)	60.48 (7.72)	60.98 (10.38)	0.96
Sex					0.32
Male	64 (42.4)	20 (40.8)	17 (35.4)	27 (50.0)	
Female	87 (57.6)	29 (59.2)	31 (64.6)	27 (50.0)	
Marital status					0.10
Married or cohabiting	118 (78.1)	36 (73.5)	39 (81.3)	43 (79.6)	
Single	33 (21.9)	13 (26.5)	9 (18.7)	11 (20.4)	
Education					0.56
Primary	23 (15.2)	8 (16.3)	6 (12.5)	9 (16.7)	
Secondary	94 (62.3)	28 (57.1)	34 (70.8)	32 (59.3)	
Tertiary or above	34 (22.5)	13 (26.5)	8 (16.7)	13 (24.1)	
Body mass index, mean (SD)	22.35 (3.57)	22.86 (3.17)	21.10 (3.44)	22.95 (3.81)	0.22
KPS, mean (SD)	89.14 (7.57)	89.18 (8.62)	88.96 (6.92)	89.26 (7.23)	0.98
Months since diagnosis, mean (SD)	26.70 (32.59)	28.06 (39.90)	24.41 (35.33)	25.70 (19.01)	0.09
Treatment modalities					0.26
Targeted therapy	94 (62.3)	35 (71.4)	29 (60.4)	30 (55.6)	
Non-targeted therapy	54 (35.8)	13 (26.6)	17 (35.4)	24 (44.4)	
Chemotherapy	33 (21.9)	9 (18.4)	8 (16.7)	16 (29.6)	
Other treatment	21 (13.9)	4 (8.2)	9 (18.7)	8 (14.8)	
No treatment	3 (2.0)	1 (2.0)	2 (4.2)	0 (0.0)	
Smoking behavior					0.36
Yes	9 (6.0)	1 (2.0)	4 (8.3)	4 (7.4)	
No	142 (94.0)	48 (98.0)	44 (91.7)	50 (92.6)	
Drinking behavior					0.88
Yes	13 (8.6)	5 (10.2)	4 (8.3)	4 (7.4)	
No	138 (91.4)	44 (89.8)	44 (91.7)	50 (92.6)	
Employment					0.60
Employed	30 (19.9)	12 (24.5)	8 (16.7)	10 (18.5)	
Unemployed	121 (80.1)	37 (75.5)	40 (83.3)	44 (81.5)	
Financial hardship					0.06
Yes	40 (26.5)	12 (24.5)	8 (16.7)	20 (37.0)	
No	111 (73.5)	37 (75.5)	40 (83.3)	34 (63.0)	
Difficulty concentrating^#^					0.64
Yes	80 (53.0)	24 (49.0)	28 (58.3)	28 (51.9)	
No	71 (47.0)	25 (51.0)	20 (41.7)	26 (48.1)	
Difficulty remembering^#^					0.53
Yes	132 (87.4)	43 (87.8)	40 (83.3)	49 (90.7)	
No	19 (12.6)	6 (12.2)	8 (16.7)	5 (9.3)	

Abbreviation: *SD*, standard deviation

^#^Scored at least 2 in the respective item in the cognitive functioning scale of the European Organization for Research and Treatment of Cancer Quality of Life Core Questionnaire

### Effect of the intervention on cognitive function

Table 2 presents the results from the mixed-model analysis. The improvement in the Tai Chi group was significantly greater compared to the control group at T1 (between-group difference, 8.34; 95% CI, 5.05, 11.63; *P* < 0.001) and T2 (between-group difference, 5.94; 95% CI, 2.09, 9.78; *P* < 0.001). The improvement in the Tai Chi group was also significantly greater compared to aerobic exercise at T1 (between-group difference, 6.52; 95% CI, 3.26, 9.79; *P* < 0.001) and T2 (between-group difference, 3.55; 95% CI, 0.04, 7.06; *P* = 0.05). In contrast, no significant improvement was observed in the aerobic exercise group compared to the control group at T1 (between-group difference, 1.82; 95% CI, − 1.33, 4.97; *P* = 0.50) and T2 (between-group difference, 2.39; 95% CI, − 1.41, 6.18; *P* = 0.40). An additional analysis was performed, adjusting for the recruitment site as a factor, and the results remained consistent and unchanged.

**Table 2:** Mixed-model analysis

	Between-group difference						Overall *P*-value
	Aerobic-control		Tai Chi-control		Tai Chi-aerobic		
	Estimate (95% CI)	*P*-value	Estimate (95% CI)	*P*-value	Estimate (95% CI)	*P*-value	
T1-T0	1.82 (− 1.33, 4.97)	0.50	8.34 (5.05, 11.63)	< 0.001*	6.52 (3.26, 9.79)	< 0.001*	< 0.001*
T2-T0	2.39 (− 1.41, 6.18)	0.40	5.94 (2.09, 9.78)	< 0.001*	3.55 (0.04, 7.06)	0.05*	< 0.001*

*T0*, baseline; *T1*, post-intervention (16 weeks); *T2*, 8 months post-intervention (12 months)

^*^P < 0.05

### Mediation analysis

Table 3 shows the mediation analyses of the relationship between the Tai Chi and control groups. Anxiety at month 4 explained 23% of the intervention effect on cognitive function at month 12. Sleep disturbance, fatigue, anxiety, and depression at month 12 explained 24%, 31%, 32%, and 24% of the effect of the intervention on cognitive function at month 12, respectively.

**Table 3:** Mediation analysis results

	Total effect	*P*-value	Direct effect	*P*-value	Indirect effect	*P*-value	Percentage mediated
	Effect size (95% CI)		Effect size (95% CI)		Effect size (95% CI)		
Cognitive function outcome at month 12							
Sleep disturbance at month 4	7.42 (2.37, 12.46)	0.004*	6.53 (1.55, 11.52)	0.01*	0.88 (− 0.44, 2.21)	0.19	11.9%
Sleep disturbance at month 12	7.42 (2.37, 12.46)	0.004*	5.61 (0.56, 10.66)	0.03*	1.80 (0.06, 3.55)	0.04*	24.3%
Fatigue at month 4	7.42 (2.37, 12.46)	0.004*	6.54 (1.35, 11.73)	0.01*	0.88 (− 0.57, 2.32)	0.23	11.8%
Fatigue at month 12	7.42 (2.37, 12.46)	0.004*	5.13 (0.39, 9.88)	0.03*	2.28 (0.11, 4.46)	0.04*	30.8%
Anxiety at month 4	7.42 (2.37, 12.46)	0.004*	5.69 (0.63, 10.75)	0.03*	1.73 (0.02, 3.43)	0.05*	23.3%
Anxiety at month 12	7.42 (2.37, 12.46)	0.004*	5.06 (0.10, 10.03)	0.05*	2.35 (0.37, 4.34)	0.02*	31.7%
Depression at month 4	7.42 (2.37, 12.46)	0.004*	6.52 (1.45, 11.59)	0.01*	0.89 (− 0.38, 2.17)	0.17	12.1%
Depression at month 12	7.42 (2.37, 12.46)	0.004*	5.68 (0.85, 10.50)	0.02*	1.74 (0.17, 3.65)	0.04*	23.5%

*p < 0.05

## Discussion

To the best of our knowledge, this is the first and largest secondary analysis of a three-arm randomized controlled trial to explore the head-to-head comparative effects of aerobic exercise and Tai Chi with a self-managed control group on cognitive function in advanced lung cancer survivors with perceived cognitive impairment. In the parent trial, both aerobic exercise and Tai Chi interventions demonstrated significant improvements in sleep disturbances and associated symptoms such as anxiety and depression, which are known risk factors of cognitive impairment [27]. Notably, our findings showed that Tai Chi improved cognitive function at T1 and T2 compared with the aerobic exercise and control groups. The improvements in cognitive functioning have reached the minimally important differences of at least 3 points on the cognitive functioning scale of the EORTC QLQ- C30 in the advanced cancer population [37, 38]. This result further suggests that improvement in cognitive function may manifest through positive effects on early changes in anxiety and later changes in sleep disturbances, fatigue, anxiety, and depression.

In contrast to the insignificant effects observed in the aerobic exercise group, the Tai Chi group exhibited greater improvements in cognitive function, including perceived concentration and memory impairment, at T1 and T2 than the aerobic exercise and control groups. This finding aligns with a comprehensive review that showed Tai Chi had a positive effect on the improvement of cognitive function in older adults with cognitive impairment [39]. Unlike the gradual progression in healthy older adults, cognitive impairment in advanced lung cancer patients tends to be further exacerbated by a combination of physical and psychological symptoms, such as compromised lung function and fatigue [8, 40]. Moreover, the neurotoxic effects of cancer treatments pose an additional risk for a significant decline in cognitive function among these patients [8]. Notably, the enhancement in cognitive function persisted until 8 months after the completion of the intervention, suggesting a sustained effect on outcomes due to residual gains from residual gains from the 16-week Tai Chi intervention. Therefore, continued practice of Tai Chi can prevent cognitive decline and improve cognitive function. Tai Chi differs from conventional aerobic exercise because it is a multimodal, low-intensity, mind–body exercise that integrates deep breathing, slow and flowing movements, sustained attention focusing, and meditation [41]. Practicing meditation, an essential component of Tai Chi, has been shown to reshape the patterns of brain structure and functional connectivity [42, 43]. Specifically, practicing Tai Chi can improve the connectivity between the prefrontal, motor, and occipital cortex, affecting myogenic activity, the sympathetic nervous system, and endothelial cell metabolic activities [44]. These changes can enhance functional brain connections and contribute to the ability of Tai Chi to improve cognition and mitigate memory decline, even within a relatively short period, such as 3 months. Consequently, repeated practice of Tai Chi can induce reliable and optimized changes in brain anatomy and function [44].

This study is the first to show that the cognition-improving effects of Tai Chi are potentially mediated by reduced anxiety, depression, fatigue, and sleep disturbance. These findings add to our understanding of the therapeutic effects of Tai Chi on cognitive function and suggest potential psychoneurological symptom-mediating pathways to explain the Tai Chi-cognitive relationship. The etiology of cancer-related cognitive impairment may be explained by exposure to prolonged psychological stressors that overload the patients’ neurological allostasis caused by cancer, its treatment, and psychological distress [45]. The resulting changes in neural biology lead to hypothalamic–pituitary–adrenal axis dysregulation and subsequent cognitive changes [45]. Hypothalamic–pituitary–adrenal axis dysregulation can result in multiple psychoneurological symptoms, such as sleep disturbances, fatigue, anxiety, and depression in patients with cancer [46]. The significant potential mediation finding suggests that psychoneurological symptoms play an underlying role in cognitive changes in these patients. The unique component of mindfulness meditation practice in Tai Chi targets multiple emotional and cognitive processes by decreasing emotional reactivity [47, 48] and attentive thoughts [49], enhancing the unprejudiced reappraisal of arrestive experiences, and downregulating the stress response through the hypothalamic–pituitary–adrenal axis and sympathetic nervous system [13], all of which can reduce the severity of psychoneurological symptoms and influence several cognitive functions [50, 51]. Notably, mitigating anxiety appears to be essential in the early improvement of cognitive impairment, whereas additional symptoms such as depression, sleep disturbances, and fatigue are key factors in the later stages. Future interventional studies could potentially target anxiety and other psychoneurological symptoms when treating patients with advanced lung cancer and cognitive impairment. Furthermore, additional adequately powered studies are warranted to further investigate the potential mechanism of mediation in this context.

This study had several limitations. First, cognitive function was measured using self-reported items from a quality-of-life questionnaire that merely targets concentration and memory, potentially underestimating an individual’s other cognitive symptoms such as language, thinking, and executive function. Future studies should adopt a standard cancer-specific questionnaire developed specifically to measure cognitive function, such as the FACT-Cog, which may more comprehensively capture cancer-related cognitive impairments. Secondly, objective testing, such as an objective neuropsychological test battery recommended by the International Cancer Cognition Task Force, should be added to strengthen the rationale for an observed benefit [52]. Thirdly, self-reported outcomes may be subject to social desirability bias, which may limit the accuracy of the results. Fourthly, this analysis was not planned a priori, and caution is warranted in interpreting the findings. Fifthly, participants in the two interventions seemingly have asymmetric exposure. However, we addressed this issue by implementing weekly WhatsApp reminders to compensate for the differences in exposure. It is important to highlight those participants in the aerobic exercise group, on average, nearly achieved the recommended level of exercise. Lastly, we acknowledged the relatively high attrition rate in the study. However, it is worth mentioning that 64% of those who dropped out were deceased at 1-year follow-up, representing a natural disease progression.

## Conclusion

Tai Chi was more effective in reducing cognitive impairment than aerobic exercise in advanced lung cancer survivors with perceived cognitive impairment. The 16-week Tai Chi intervention demonstrated significant positive effects on cognitive function, indicating that Tai Chi can be a complementary therapeutic option for advanced lung cancer survivors and cognitive impairment. Anxiety, depression, fatigue, and sleep disturbances mediate the relationship between Tai Chi and cognitive function, with anxiety playing a significant role at an earlier stage. Therefore, early treatment to alleviate anxiety may be a promising approach for the early management of cognitive impairment in advanced lung cancer survivors. These results highlight the potential of Tai Chi as a safe and complementary therapeutic option for managing cognitive impairment in this vulnerable population. In addition, evaluating the effects of improving psychoneurological symptoms on promoting cognitive function in cancer survivors with cognitive impairment is of great research interest. Future interventions targeting cognitive impairment should address psychoneurological symptoms to optimize this effect.

## Data Availability

No datasets were generated or analyzed during the current study.
